# Supplementary material for: Safety and efficacy of colchicine in COVID-19 patients: A systematic review and meta-analysis of randomized control trials
Source: PLoS One. 2022 Apr 5;17(4):e0266245. doi: 10.1371/journal.pone.0266245 (PMC8982874; doi:10.1371/journal.pone.0266245)
Supplement: S1 Table — (DOCX) [file pone.0266245.s002.docx]

**Online Supplementary**

**Table S1: Detailed search strategy.**

| **PubMed (141)** | ("colchicine"[MeSH Terms] OR "colchicine"[All Fields] OR "colchicin"[All Fields] OR "colchicines"[All Fields] OR "colchicine s"[All Fields]) AND ("covid 19"[All Fields] OR "covid 19"[MeSH Terms] OR "covid 19 vaccines"[All Fields] OR "covid 19 vaccines"[MeSH Terms] OR "covid 19 serotherapy"[All Fields] OR "covid 19 serotherapy"[Supplementary Concept] OR "covid 19 nucleic acid testing"[All Fields] OR "covid 19 nucleic acid testing"[MeSH Terms] OR "covid 19 serological testing"[All Fields] OR "covid 19 serological testing"[MeSH Terms] OR "covid 19 testing"[All Fields] OR "covid 19 testing"[MeSH Terms] OR "sars cov 2"[All Fields] OR "sars cov 2"[MeSH Terms] OR "severe acute respiratory syndrome coronavirus 2"[All Fields] OR "ncov"[All Fields] OR "2019 ncov"[All Fields] OR (("coronavirus"[MeSH Terms] OR "coronavirus"[All Fields] OR "cov"[All Fields]) |
| --- | --- |
| **Cochrane Central (53)** | (colchicine) AND (covid-19 OR SARS-CoV-2 OR coronavirus) |
| **PMC NCBI (956)** | ("colchicine"[MeSH Terms] OR "colchicine"[All Fields]) AND ("COVID-19"[All Fields] OR "COVID-19"[MeSH Terms] OR "COVID-19 Vaccines"[All Fields] OR "COVID-19 Vaccines"[MeSH Terms] OR "COVID-19 serotherapy"[All Fields] OR "COVID-19 Nucleic Acid Testing"[All Fields] OR "covid-19 nucleic acid testing"[MeSH Terms] OR "COVID-19 Serological Testing"[All Fields] OR "covid-19 serological testing"[MeSH Terms] OR "COVID-19 Testing"[All Fields] OR "covid-19 testing"[MeSH Terms] OR "SARS-CoV-2"[All Fields] OR "sars-cov-2"[MeSH Terms] OR "Severe Acute Respiratory Syndrome Coronavirus 2"[All Fields] OR "NCOV"[All Fields] OR "2019 NCOV"[All Fields] OR (("coronavirus"[MeSH Terms] OR "coronavirus"[All Fields] OR "COV"[All Fields]) |
