# Supplementary material for: Safety and efficacy of colchicine in COVID-19 patients: A systematic review and meta-analysis of randomized control trials
Source: PLoS One. 2022 Apr 5;17(4):e0266245. doi: 10.1371/journal.pone.0266245 (PMC8982874; doi:10.1371/journal.pone.0266245)
Supplement: S2 Table — (DOCX) [file pone.0266245.s003.docx]

**Online Supplementary**

**Table S2: Baseline characteristics of patients included in each study.**

| **Author, year** | **BMI median** | | **C-reactive protein mg/L** | | **D-dimer, μg/ml** | | **Glucose mmol/L** | | **Creatinine, μmol/l** | | **Any oxygen support, n (%)** | |
| --- | --- | --- | --- | --- | --- | --- | --- | --- | --- | --- | --- | --- |
|  | **Treatment** | **Control** | **Treatment** | **Control** | **Treatment** | **Control** | **Treatment** | **Control** | **Treatment** | **Control** | **Treatment** | **Control** |
| **Mareev et al, 2021** | Mean 30.2 (3.59) | 30.6 (5.37) | 99.4 [57.7; 116] | 91.5 [59.2; 131] | 0.87 [0.58- 1.24] | 1.12 [0.79- 1.37] | Mean 5.74 (1.03) | 6.05 (0.81) | 89.3 (20.5) | 86.6 (25.0) | 14 (66.7) | 12 (54.5) |
| **Holby et al, 2021** | N/A | N/A | N/A | N/A | N/A | N/A | N/A | N/A | N/A | N/A | 1795 (32) | 1768 (31) |
| **Tardif et al, 2021** | 30·0 (6·2) | 30·0 (6·3) | N/A | N/A | N/A | N/A | N/A | N/A | N/A | N/A | N/A | N/A |
| **Deftereos et al, 2020** | 27.7 (24.6 to 30.5) | 27.3 (25.2 to 30.5) | 4.0  (1.2 to 9.5) | 3.6 (1.0 to 6.7) | 0.60  [0.40-1.01] | 0.52 [0.28- 0.94] | Median 106 (91-126) | 100 (88-125) | N/A | N/A | N/A | N/A |
| **Lopes et al, 2021** | 29.7 (26.3–36.0) | 33.5 (28.6–37.8) | 9.2 (6.6–12.6) | 9.3 (5.8–15.1) | 1.12 [0.63-1.77] | 1.40 [0.92-1.92] | N/A | N/A | 0.90 (0.70–1.01) | 0.81 (0.64–1.05) | 28 (78) | 24 (67) |
