# Supplementary material for: Safety and efficacy of colchicine in COVID-19 patients: A systematic review and meta-analysis of randomized control trials
Source: PLoS One. 2022 Apr 5;17(4):e0266245. doi: 10.1371/journal.pone.0266245 (PMC8982874; doi:10.1371/journal.pone.0266245)
Supplement: Attachment — Submitted filename: Rebuttal Letter.docx [file pone.0266245.s004.docx]

January 10^th^, 2022

Editor-in-Chief

PLOS-One

Dear Editor,

We are pleased to submit our revised manuscript entitled ‘Safety and efficacy of colchicine in COVID-19 patients: A systematic review and meta-analysis of randomized control trials’ for consideration for publication in PLOS-One.

We appreciate the comments we received from the editors and reviewers, all of which have been addressed in this revised version of the paper. Please find below the editor’s and reviewer’s comments in bold and the author responses. We further ensure that the material has not and will not be offered elsewhere for possible publication, as it is under consideration.

We hope that these changes will make the manuscript competitive for publication in the journal.

**Salim Surani,** MD, MPH, MSHM, FACP, FCCP, FAASM

Adjunct Clinical Professor of Medicine and Pharmacology,

Texas A&M University, USA.

Clinical Professor, University of Houston (Voluntary

Email: [salimsurani@gmail.com](mailto:salimsurani@gmail.com)

**You state: The strengths of the study are not clear, and it should be highlighted more as to what this study adds to the medical literature and how they address the current gap.**

Response: Thank you so much for pointing this out. We have now edited the manuscript to include the following details:

This study has several strengths. Firstly, it includes all gold-standard RCTs that largely prove the safety and efficacy of Colchicine as an anti-inflammatory medication for different inflammatory conditions, including COVID-19. We have also included the findings of recent large-scale RECOVERY trial that contributed to the estimates in our study, and concluded that Colchicine has no significant effect on all-cause mortality and mechanical ventilation. Hence, our study expands the findings of prior literature by including contemporary trials that were missed by previous meta-analyses. The pooled estimates of prior meta-analyses were largely driven by non-randomized observational data leading to conflicting findings, the conclusion of our meta-analysis (based on only RCTs) bring consensus on the utility of Colchicine in these patients. Other reason being all included studies had some concern for risk of bias in all previous meta-analyses. Secondly, our meta-analysis comprising of only RCTs includes a summary of 16,048 COVID-19 positive patients indicating higher statistical power in comparison to previous meta-analyses. Lastly, we have demonstrated a higher methodological quality of the included trials using the standard recommended ROB-2 approach, to ensure the quality of data for inclusion of studies in our meta-analysis model. Given this, we believe our large-scale meta-analysis could serve as a benchmark against which the findings of individual studies and future trials could be compared.

**You state: The limitations may be somewhat enhanced further by highlighting additional limitations that resulted in study heterogeneity or why subgroup analysis was not performed.**

Response: Thank you so much for pointing this out. We have now edited the manuscript to include the following details:

Different dosage and the duration of the loading dose and overall length of therapy presents as a limitation of this study. Due to the low number of studies per outcome and limited available data, a sensitivity analysis was performed in place of subgroup analysis to assess high heterogeneity.

**You state: Overall the quality of discussion is commendable, but it needs to proofread for example the capitalization error in line 281.**

Response: Thank you for pointing this out. The manuscript has been proof-read and corrected for grammatical and spelling errors.

**You state: It can enhance the quality of results further if the number of studies that were included for mechanical ventilation and all-cause mortality was mentioned at the beginning of the paragraph.**

Response: Thank you so much for pointing this out. We have now edited the manuscript to include the following details:

All-cause mortality was reported in all five studies included.

And

Of the four studies included in quantitative analysis, there was no statistically significant reduction in mechanical ventilation.
